# Factors contributing to incivility amongst students at a South African nursing school

**DOI:** 10.4102/curationis.v38i1.1464

**Published:** 2015-10-07

**Authors:** Hildeguard Vink, Oluyinka Adejumo

**Affiliations:** 1 School of Nursing, University of the Western Cape, South Africa

## Abstract

**Background:**

This study determined the experiences of nurse educators of the factors contributing to the uncivil classroom behaviours of nursing students at a South African school of nursing.

**Objective:**

To describe what nurse educators consider to be factors contributing to incivility among nursing students in a South African nursing school.

**Method:**

A qualitative descriptive design was used. Eleven nurse educators were purposively sampled for their experiences on the factors contributing to incivility. Individual face-to-face interviews were conducted until data saturation.

**Results:**

The data analysed indicated that the educators had varying but often similar perspectives on which factors contribute to incivility among nursing students. The three themes that emerged from the data were academic, psycho-pathological and social factors. The themes were discussed on the basis of their reported impact on classroom behaviour and the implications for the teaching and learning environment.

**Conclusion:**

Conclusions were made that an educational screening system to identify committed students before admission into nursing education should be explored; that a support system should be explored for nurse educators to deal with incidents of uncivil behaviour, perhaps within policy frameworks in the nursing institution; that emotional support should be provided for students who may be experiencing difficulties adjusting to the rigours of post-secondary education; and that a forum should be set up for nurse educators to compare notes and share ideas on what works best in reducing the incidence of uncivil behaviours in the classroom setting.

## Introduction

In nursing and nursing education incivility is becoming more visible in nursing schools and classroom climates that are unpleasant and unco-operative are becoming the norm in higher education (Patron & Bisping 2008:61). Incivility in the classroom is regarded as a concept that is very difficult to define because it is based on individual perceptions. It is therefore important to understand that people attach different values and meanings to incivility – what one educator perceives as uncivil may not be deemed as such by another (Bjorklund & Rehling 2010:15).

Clark, Farnsworth and Landrum (2009:7) defined incivility in nursing education as ‘rude or disruptive behaviours which often result in psychological or physiological distress for the people involved and if left unaddressed, may progress into threatening situations’. Incivility mostly seems to be verbal in nature but could also occur through electronic communication devices. Students’ behaviour is a concern for nursing faculty as nursing is a profession and students become professional through socialisation into the profession in the classroom and clinical practice (White 2011:43).

However, despite faculty raising concerns about students not showing courtesy, acting disrespectfully and being disruptive, very little research has been conducted on classroom incivility in higher education (Ausbrooks, Jones & Tijerina 2011:255). Clark and Springer (2010:319) and Luparell (2004:59) also raised concerns that very little empirical data exists on incivility in nursing education. Luparell (2011:93) wondered whether perhaps something in the healthcare environment creates a platform for incivility – or is it just that some individuals have a natural tendency towards uncivil behaviour and continue exhibiting such behaviour throughout their nursing study programme.

Luparell (2007:19) is of the opinion that a lot of work needs to be done to understand the phenomenon of incivility. She stated further that an exploration of the potential implications that student incivility can have on the nursing profession is needed if students who act uncivilly towards their nurse educators are allowed into the nursing profession. More discourse is also needed on basic nursing values and the nurse educators’ role in building the nursing profession (Luparell 2007:19).

According to Delucchi and Korgen (2002:103–104), students in higher education act more as consumers these days, and therefore have a sense of entitlement since they are paying tuition fees: 42.5% of sociology major students they surveyed agreed that ‘If I’m paying for my college education, I’m entitled to a degree’. This implies that students want high marks with little effort for the money they pay and that they also expect educators to hold their attention in class. Students described in a consumerist academic environment do not think that higher education includes ‘effort, challenge, or constructive criticism’ (Delucchi & Korgen 2002:101).

Unfortunately, in the teaching-learning process it is unavoidable for the educator in higher education not to deal with or comment on the performance, conduct and behaviour of a student, since that student is in a learning process. The performance of a student should be equal to or higher than the standards set for academic achievement; their conduct is subject to ethical codes, and their behaviour is determined by the rules of behaviour expected and set by the educator and the institution. However, it is often issues of performance assessment and student conduct in class that are responsible for conflict between teacher and student (Miller 2009:248).

Factors such as large group size have been identified as a possible contributor to academic incivility amongst accounting students (Elder, Seaton & Swinney 2010:96). Clark and Springer (2010:321–322) found managing various roles related to work, studies and family, financial burdens, time-management issues, lack of support and incivility from faculty, and personal or mental health issues are stressors that may contribute to incivility. However, acts of incivility may sometimes occur without any prior event or warning (Luparell 2004:61). Nurse educators, according to Williamson (2011:66), reported experiencing strong emotions in response to student incivility – such as surprise when being caught off guard.

It is also believed that educators may be at the root of classroom incivility, as they might lack the necessary training in classroom management to keep the students engaged in learning activities. Poor teaching and classroom management coupled with anonymity in large-group teaching settings make it even more difficult for educators to keep students engaged. This could be a source of frustration and dissatisfaction to them and to those students who are keen learners (Newman-Gonchar 2002:63). It is therefore important that the weakened relationships between students and their educators should be examined and programmes and measures to increase civility on the campus should be developed in order to respond to the changing nature of campuses (Newman-Gonchar 2002:62).

In South Africa, whilst a lot of casual discussions exist amongst nurse educators about the incidents and their experiences with nursing students’ classroom incivility, factors contributing to incivility amongst nursing students have not been reported on in the literature. Moreover, whilst it is acknowledged that acts of incivility exist in South African nursing schools, very little is known about the possible causes of the phenomenon of nursing students’ classroom incivility in them.

This article reports on the experiences of nurse educators of the factors contributing to uncivil classroom behaviours of nursing students in a South African school of nursing. The research question used to guide the data collection is: From their own experience with nursing students, what are the experiences of nurse educators’ of the factors contributing to incivility amongst students at a South African school of nursing.

## Methodology

### Design

A qualitative methodology using a descriptive research design was adopted for this study.

### Research setting and population

The research setting was a nursing school in South Africa that offers both undergraduate and postgraduate nursing education. This particular nursing school was selected because of the large number of nurse educators employed there and its representativeness of the South African racial groups. In addition, it is one of the settings that had been identified in the past for acts of incivility amongst the students. The study population consisted of full-time nurse educators employed at the main campus of this nursing school teaching undergraduate nursing students; 25 educators qualified to be included in the study. The nurse educators’ experience in nursing education varied from approximately two to 27 years, and most of them had vast experience in teaching nursing students at undergraduate level. Educators also had experience derived from working at other nursing schools.

### Sample and sampling process

Eleven nurse educators with vast experience of the phenomenon being studied were purposively sampled until data saturation was reached (Creswell 2009:178).

### Data collection and analysis

Individual face-to-face, audio-recorded interviews were conducted with each participant at a time and place agreed upon by the participant. An interview guide was used, with one main trigger question requesting each participant what they consider as factors contributing to incivility based on their experiences with nursing students in a classroom. All the interviews were conducted over a period of three weeks; each interview lasted between 15 and 40 minutes and was transcribed verbatim. Probing was done for clarification purposes. In order to obtain the best results, it was necessary to maintain the natural setting in which the problem occurred (Streubert & Carpenter 2007:28).

Data analysis started after data saturation was reached. Familiarisation with the data was achieved by carefully listening to the audio-recordings and repetitive reading of the individual participants’ transcripts (Streubert & Carpenter 2007:69). The researchers and an independent coder assigned codes to the meanings of phrases or sentences from the transcribed interviews. The transcripts were considered one at a time in order to identify relevant units of meaning in the form of words, phrases, sentences and paragraphs. These were converted into codes that were later classified as themes, categories and patterns. The extracted codes were compared with the independent coder's and where differences occurred these were resolved by further examination and analysis of the transcribed texts.

When all of the relevant units of meaning were identified from all of the interview transcripts, the researchers copied all the related units of meaning onto a spreadsheet under the relevant themes. The organisation of data under the themes help-ed the researchers to look more closely at the data under each theme and to identify whether any parts were missing, what the commonalities were, what the uniqueness in content was or whether there was any confusion and contradiction (Streubert & Carpenter 2007:36, 62). Once the researchers were satisfied that the material under each theme was relevant, categories were formulated and after that the researchers looked at recurrent experiences in the participants’ responses in order to identify emerging patterns.

The main goal was to produce accurate descriptions based on the research question, and therefore quotations, comments and stories from the original data gathered from the different participants’ transcripts were used (Streubert & Carpenter 2007:23). This was a way of ensuring that the findings related accurately to the phenomenon under study, and assisted in the elimination of bias from the researchers. The researchers also made use of bracketing to minimise bias (Connelly 2010:127).

## Ethical considerations

Permission to conduct the study was granted by the nursing school's interim Research Committee after approval of the methodology and the ethics of the research project. The participants received written information about the purpose of the study and how they would be protected during the research, after which they indicated their interest in participating in the study or not. Informed consent was obtained prior to each interview, after participants’ understanding of the information described on the information sheet was first checked by the researchers. Permission to audio-record the interviews was also obtained from each participant, and the reason for making handwritten notes was explained. To ensure anonymity, a code was assigned to each participant's interview protocol and the same code was used to identify the individual participant's audio-recording. Each participant had been informed that taking part was voluntary and that they could withdraw from the study at any time if necessary, without any penalty or consequence to them. The researchers also informed the participants that counselling services were available if needed, because of the sensitive nature of the study.

## Results

From the participants’ descriptions it became clear that nurse educators did experience incivility in the particular nursing school, and the participants cited various factors that they thought were contributing to the occurrence of the phenomenon in the nursing school, particularly in the classroom.

## Themes

Three themes were extracted from the data for the factors considered by the participants to be responsible for classroom incivility amongst the students: Theme 1 – Academic factors, with academic setting, students’ academic preparedness, nurse educators’ attitudes or behaviour, and the influence of the Students’ Repesentative Council (SRC) as sub-themes; Theme 2 – Psycho-pathological factors, with sub-themes emotional disturbances and substance abuse; Theme 3 – Social factors, with cultural and social issues as a sub-theme. These are described in further detail below.

### Theme 1: Academic factors

Academic-related factors associated with uncivil behaviour on the part of students in the classroom included the academic setting, students’ academic preparedness, nurse educators’ attitudes or behaviour, and the influence of the SRC.

### Sub-theme 1.1: Academic setting

The participants regarded large group size in the nursing school to be the major contributor to student incivility. They stated that such an environment provides students with anonymity, which may contribute to incivilities that are not conducive to the teaching and process. Nurse educators build their experiences with a large group size upon their experience of teaching smaller numbers of students. Whilst classroom environments with large student totals evoked feelings of anxiety and a lack of control, the smaller classes with fewer students were reported to provide for a more positive experience (Tilley 2014:66–67). Nurse educators also believed that students’ concentration and attention were likely to be affected in classroom environments with large student totals, because the lecture as a teaching method seems to be the only effective way for the teachers to transfer knowledge to larger student groups. The nurse educators also believed the teaching of too many students in a class to be a potential source of discomfort for students:

‘Also our classrooms are extremely big at the nursing school and I think that is also a big cause for the bad behaviour in class. So a relationship between the student and the lecturer has been built nicely in a smaller classroom setting. However, in the big classroom at the end of the year you still say the name and you don’t have a face to the name and it's just too much and I think that impacts.’ (Participant 8)

### Sub-theme 1.2: Students’ academic preparedness

The participants also asserted that students are challenged by an academic environment that requires higher standards than what their previous school backgrounds catered for. Students’ preparation for academia had been from a different teaching approach to what they were exposed to in the nursing school. The students concerned were also observed to be displaying distracting behaviours, which had an impact on students who wished to learn. The participants believed that the nursing curriculum is overloaded with content and that students cannot possibly cope; they therefore become frustrated in their quest to succeed. The participants explained that students become desperate and commit fraud in their examinations and assessments:

‘I think we getting students who just don’t have the academic skills and I think it's really, really frustrating. I think they are challenged by requirements that are beyond their abilities, their current abilities, and I know we are doing everything to try and improve it.’ (Participant 5)

‘But for fraud I think there are quite a lot of things that's adding towards it and I’ve mentioned this elsewhere, where we ignore the National Benchmark Test (NBT) result that indicates that the actual students we accept here have got very little chance of succeeding in this programme, right. I think it plays an important role here in this fraud. Because this fraud is not just signing fraudulently for the attendance register, it goes into the exams as well; it goes into the tests as well.’ (Participant 7)

### Sub-theme 1.3: Nurse educators’ attitudes or behaviour

Nurse educators’ openness in identifying that they themselves could contribute to student incivility was worthy of note in this study. They especially became aware of this as they acknowledged the diversity of students that they interact with daily. They reported that there might be a divide between students and educators, as students might perceive the latter to be unapproachable, abrupt and sometimes even unfair:

‘Then also it can also be the attitude of the lecturers, because … Hey, yes it can be because I think sometimes lecturers can also be very abrupt and you know behaviour breeds behaviour.’ (Participant 3)

### Sub-theme 1.4: Influence of the SRC

The role that SRC members played in instigating and participating in classroom incivility, such as late-coming, unpreparedness and fraud, was reported by participants to be worrisome, as it had an impact on classroom behaviour. The SRC members were also described by the participants as lacking regard for authority figures, and acting like a worker union. The participants described the SRC as powerful and perceived its members to have more rights than the educators, leaving the educators with the feeling of a lack of support from the nursing school management:

‘They talk to each other so a lot of things go via the SRC and so on so they get to know and quite often it's some of the SRC members who are the difficult ones; it's almost like they won’t take instructions from anybody. They walk in and out, some students get to class at 9 o’clock, and they come in with a diary.’ (Participant 1)

### Theme 2: Psycho-pathological factors

From the psycho-pathological factors described by the participants two sub-themes – emotional disturbances and substance abuse – emerged as factors associated with uncivil behaviour by students in the classroom.

### Sub-theme 2.1: Emotional disturbances

The study found that students experience anxiety and frustration as well as, particularly, fear of failure in a programme with high demands and standards, where they struggle to communicate their needs These are some of the emotional factors from the nurse educators’ perspectives that could lead to uncivil behaviour:

‘I think there is a high level of frustration, of anxiety amongst the students, even fear, which leads to some of the uncivil behaviour.’ (Participant 5)

### Sub-theme 2.2: Substance abuse

Signs of substance abuse amongst students have also been reported. Although cited by very few participants, it is, however, considered as worthy of note in this study. Students’ behaviour was related as being noisy and restless, and that they lacked honesty:

‘What triggers this noisy behaviour and things like this. A lot of the students come into class under the influence of alcohol. You can see they sit with their ‘Tik’ [*crystal methamphetamine*] eyes in there, especially they get restless before tea and they get restless here before lunch, and you can actually see how their behaviour changes.’ (Participant 6)

### Theme 3: Social factors

Cultural and social issues emerged as a sub-theme under the theme of social factors as the participants described how these contribute to student classroom incivility.

### Sub-theme 3.1: Cultural and social issues

Students reportedly are different today than in previous years and come from different social and cultural backgrounds, with varying norms and values. Coupled with perhaps a lack of strong role models, poor social interaction and a lack of respect for the female nurse educator also became an issue of conflict:

‘Also our students come from different cultural backgrounds or different areas where they may be, I don’t know how they have been schooled, schooled all differently coming from different areas, so the respect for the classroom is different, it varies.’ (Participant 8)

Many students enrolled in this nursing programme have been reported to come from socially and financially disadvantaged backgrounds, and therefore struggle with very basic needs:

‘Then also poverty – some people behave different under different circumstances. When they come here, they have got no money, they need toiletries, and they need food. Although they get lunch and supper, they don’t get breakfast. When they go to the services they never got a meal to take with them; it might have changed, that I’ve heard, so they don’t have money for the basic things. But I don’t think it's really necessary to be rude, but that's what I think.’ (Participant 3)

## Discussion

From the participants’ descriptions it became clear that incivility in the nursing school was attributable to various factors, particularly in the classroom. The participants linked the academic setting, with a large group size in the nursing school, to an increase in student incivility. It is believed that such environments provide students with an anonymous status. Elder *et al*. (2010:93) also hold the opinion that students may become lost in the crowd of a large classroom, and the anonymous status may lead to incivilities which preclude an effective learning environment.

Nurse educators probably often found it difficult to manage students’ behaviours, as they would not be able to identify who the perpetrators were due to the high student numbers per class. The findings in Elder *et al*. (2010:96) support this, as 77.7% of survey respondents believed that incivility is more likely to occur in a class with a large number of students as opposed to one with a smaller number.

Nurse educators were able to compare and identify differences in their experiences with larger student totals in contrast to their experiences of teaching smaller student groups. Anxiety and a lack of control were reportedly associated with a larger group size, whereas the smaller group size provided a more positive climate for teaching and learning. The lecture, as the most effective teaching method, was reported to be used in these larger classroom environments, which seems to have led to students’ becoming detached from the learning process (Cooper & Robinson 2000:6). As students lose their concentration and attention in a crowded environment, which might have also contributed to personal discomfort, incivility seems to have increased from the nurse educators’ perspectives. Tilley (2014:66–67) found that larger group size was the major contributing factor to increased complaints about student and faculty behaviour, and since class totals have been below 30 a more positive classroom atmosphere has been experienced and complaints have also decreased.

The challenges experienced by students due to high academic requirements and standards with a demanding nursing curriculum can be attributed to their previous school backgrounds that perhaps did not cater for their needs in an institution of higher education. This is believed to be the reason why students struggle to cope, and why they are reported to experience anxiety, frustration and fear. They possibly resort to activities that are regarded as fraudulent and distracting to the educators and other students. The nursing faculty in Gazza's study (2009:222) asserted that nursing students have health problems and seem to lack professionalism, and others have academic deficiencies. One faculty member commented that ‘These kids can’t write. They are not prepared for college coming out of high school’ (Gazza 2009:222). The findings also pointed out that students experience emotional responses that are possibly associated with a nursing programme that has too heavy a theoretical component, which makes them come across as uncivil.

This study showed that incivility from nurse educators could be experienced by students as nurse educators’ related awareness that they themselves could be perceived as uncivil, especially as they work with diverse groups of students. From this it is clear that there are perhaps power differences between educators and students that can be the triggers for uncivil behaviour in class. Clark and Springer (2007:96) also found that students complain that nurse educators act uncivilly towards them. Such behaviour from nurse educators may be destructive towards the student and the academic environment, and learning can be compromised as a consequence (Clark 2008:5). It is important to remember that the student is at the centre of the teaching and learning environment, and effective strategies need to be applied to foster civility.

The reported role that the SRC members played in classroom incivility and the disregard they demonstrated towards the teaching-learning environment left the educators with feelings of little control. SRCs exist in South Africa within a culture of activism: to represent students often translates into ‘fighting’ for students, even when this may be against the rules and regulations of the nursing school.

Although cited by very few participants, signs of substance abuse amongst students have also been reported, and this is considered a relevant factor that could lead to incivility, as students were described as noisy, restless and dishonest when under the influence of substances. Bjorklund and Rehling (2010:16) confirmed the presence of alcohol and drug abuse in higher education classrooms; student participants at a Midwestern public university ranked this amongst the most severe uncivil behaviours, although it occurred less frequently.

Students’ socio-economic and cultural backgrounds were also pointed out as being a contributing factor to classroom incivility. In a diverse country such as South Africa, it is to be expected that at a nursing school people from different backgrounds and belief systems will interact with one another. This interaction may lead to conflict amongst people who espouse different ideas and have had varied life experiences (Bacor 2002:12). This could even be complicated further by students’ struggling financially to fulfil basic human needs. In one study, financial pressures have been identified by faculty as one of five stressors that can contribute to student incivility (Clark & Springer 2010:321).

## Limitations

The findings in this study on factors contributing to incivility relate only to nurse educators’ perspectives from one nursing school in one province of South Africa and the sample size was small. Therefore the findings may not be generalised to similar institutions in South Africa. An exploration of incivility in South Africa that would include different nursing education institutions at different settings in this country would be needed to be able to form any generalisations on the phenomenon.

## Recommendations

Further studies on this phenomenon are required, particularly on developing effective measures to deal with the factors identified. These measures would include:

- an educational screening system to identify committed students before admission into nursing;
- exploring a support system for nurse educators to deal with incidents of uncivil behaviour, perhaps within policy frameworks in the nursing institutions;
- providing emotional support for students who may be experiencing difficulties adjusting to the rigours of post-secondary education; and
- setting up a forum for nurse educators to compare notes and share ideas on what works best to reduce the incidence of uncivil behaviours in the classroom setting.

## Conclusion

This study has assisted in bringing to the fore the perspectives of nurse educators on those factors that could be contributing to students’ uncivil behaviours in a nursing school in South Africa. Nurse educators were able to identify different circumstances which they described as contributing factors. Nurse educators or administrators who may be dealing with issues of professional nursing ethics, or requiring an understanding of the factors that contribute to uncivil behaviour amongst younger generations of student nurses in nursing schools and in the clinical setting, may benefit from the insights provided by this study.
